# A gene expression panel for estimating age in males and females of the sleeping sickness vector *Glossina morsitans*

**DOI:** 10.1371/journal.pntd.0009797

**Published:** 2021-09-23

**Authors:** Eric R. Lucas, Alistair C. Darby, Stephen J. Torr, Martin J. Donnelly

**Affiliations:** 1 Liverpool School of Tropical Medicine, Liverpool, United Kingdom; 2 Institute of Integrative Biology, University of Liverpool, Liverpool, United Kingdom; 3 Wellcome Sanger Institute, Cambridge, United Kingdom; Institut Pasteur, FRANCE

## Abstract

Many vector-borne diseases are controlled by methods that kill the insect vectors responsible for disease transmission. Recording the age structure of vector populations provides information on mortality rates and vectorial capacity, and should form part of the detailed monitoring that occurs in the wake of control programmes, yet tools for obtaining estimates of individual age remain limited. We investigate the potential of using markers of gene expression to predict age in tsetse flies, which are the vectors of deadly and economically damaging African trypanosomiases. We use RNAseq to identify candidate expression markers, and test these markers using qPCR in laboratory-reared *Glossina morsitans morsitans* of known age. Measuring the expression of six genes was sufficient to obtain a prediction of age with root mean squared error of less than 8 days, while just two genes were sufficient to classify flies into age categories of ≤15 and >15 days old. Further testing of these markers in field-caught samples and in other species will determine the accuracy of these markers in the field.

## Author summary

Many insect-borne diseases are controlled by methods that kill the insects responsible for disease transmission. Estimating the age structure of populations of disease-transmitting insects provides information on mortality rates and on the capacity of the population to maintain disease transmission, and should form part of the detailed monitoring that occurs in the wake of control programmes, yet tools for obtaining estimates of individual age remain limited. We investigate the potential of using markers of gene expression to predict age in tsetse flies, which are the vectors of deadly and economically damaging African trypanosomiases, such as sleeping sickness. We use RNA sequencing to identify candidate genes, and test their accuracy in laboratory-reared tsetse flies of known age. Measuring the expression of six genes was sufficient to obtain a prediction of age with root mean squared error of less than 8 days, while just two genes were sufficient to classify flies into age categories of ≤15 and >15 days old. Further testing of these markers in field-caught samples and in other species will determine the accuracy of these markers in the field.

## 1. Introduction

Vector-borne diseases represent major threats to health and livelihood world-wide, being directly responsible for 680,000 deaths annually [1], as well as causing huge economic damage to livestock [2,3]. Control of the vectors that transmit these diseases is an integral tool for reducing disease burden [4]. The metric of success for these control programmes is a reduction in disease burden in the host population. However, when vector control is accompanied by other interventions such as screening and treating the host population for the disease, the contribution of vector control to the subsequent reduction of disease can be hard to determine [5]. Conversely, while the impact on the vector population may not bear a simple relationship to disease burden, it is a direct outcome of vector control. Control efforts should thus be accompanied by detailed monitoring of the targeted vector populations, to estimate impact, to monitor population recovery and to understand the transmission dynamics of the disease. Mostly, monitoring currently relies on counting the number of vectors caught in sentinel traps, which can be greatly affected by trapping method, effort and efficacy, and may only partly reflect the ability of the vector population to transmit disease [6].

One aspect of vector monitoring that has been particularly challenging is the quantification of the age-distribution (demographics) of natural populations [7–9]. Estimating vector age is important for two reasons. First, it can provide a measure of the effectiveness of vector control because increased adult mortality should lead to a younger population age structure. Importantly, this measure of control effectiveness is independent of catch size and trapping effort because only the distribution of age needs to be known. Second, in most cases, the probability that an individual vector is infectious for a given disease increases with age [10,11]. Before transmitting the disease, vectors first need to have taken an infected blood meal, and there is then typically a delay between acquisition of infection and onward transmission due to the need for the pathogen to replicate and/or mature. Age grading is therefore useful to determine the proportion of individuals old enough to transmit disease.

Tsetse flies (genus *Glossina*) are the vectors of Human African Trypanosomiasis (HAT, or sleeping sickness) and Animal African Trypanosomiasis (AAT, or nagana). HAT is, without treatment, a fatal disease endemic to sub-Saharan Africa [12], while AAT presents a major economic burden to rural communities by affecting livestock [2]. Being a disease primarily of animals and with reservoirs across multiple species, AAT cannot be controlled through treatment alone and is thus highly dependent on vector control [13]. HAT can be more readily controlled through treatment of infected humans, but both the anthroponotic “Gambian” HAT and the zoonotic “Rhodesian” HAT also require some measure of tsetse control to reduce transmission [14]. The choice of tsetse control method depends largely on the ecology and feeding habits of the species being targeted, as well as on local practicalities, but most methods rely on the use of insecticides to directly kill the flies, often applied to baited targets or cattle [13], imposing increased mortality that should translate into a shift in age structure.

*G*. *morsitans morsitans* is a major vector of AAT in East and Southern Africa and can also transmit HAT [15]. Catch rates of this species in the wake of vector control can be extremely low [16–18], making it particularly challenging to conduct ongoing monitoring of important populations. It is therefore all the more important to extract as much information as possible from the limited number of flies obtained.

As is the case for all insect vectors, a means to accurately determine the age of tsetse flies is a valuable but elusive goal, and current methods have many shortcomings. Laborious ovary dissections can be used to age females up to their fourth ovarian cycle [19], but this technique requires specialist dissection skills and cannot be applied to males, despite males being at least as competent at transmission as females, and perhaps more so [15,20]. Estimates of age based on wing damage [21] or analysis of pteridines have also been used [22,23], but experience in practical applications has shown that measurements in the field vary enormously (for example in mosquitoes [24,25]) and cannot be used to reliably estimate age on an individual basis [26].

Here we explore the value of using gene expression to estimate age in tsetse flies. This method has previously been tested in mosquitoes [7,8], with encouraging results, but has yet to be applied in tsetse. We use laboratory-reared *G*. *morsitans* as a proof of concept, and show that measuring the expression of just six genes can estimate the age of both male and female tsetse flies with a root mean squared error of less than 8 days. We also trained models to classify tsetse into those younger or older than 15 days, since flies younger than 15 days are unlikely to harbour a mature trypanosome infection [15], and found that just two genes are sufficient for 95% accurate classification.

## 2. Methods

### 2.1. Sample collection and RNA extraction

*G*. *morsitans morsitans* individuals were collected from colonies maintained at the Liverpool School of Tropical Medicine. Colonies are kept in meshed boxes (cages) at 26°C ± 2°C and 72 ± 4% humidity, with a 12hr light-dark photoperiod, and fed three times per week using defibrinated horse blood (TCS Biosciences Ltd., Buckingham, UK) provided through silicon-membrane feeders. Pupae are regularly collected and allowed to emerge to form new cages. Each fly cage contains flies which eclosed over a 2–3 day window, and thus the age of all flies in the cage are known to a precision of either 2 or 3 days. The ages reported here are the middle of the age range (eg: a fly aged 13–15 days or 13–16 days is reported as 14 days old). The age of the samples ranged from 2 to 62 days. While reproductive status of females was not measured precisely, we tried to include a range of physiological states (based on visual inspection of the size of the abdomen) within each age group, so that genes could be identified that are predictive of age in spite of variation caused by the ovarian cycle. Overall, 505 flies were collected (301 female and 204 male, S1 Data).

For sample collection, fly cages were briefly transferred to a cold room (4°C) where flies to be collected were removed from the cage once quiescent and decapitated. Heads were placed into RNAlater and stored at -20°C. In case repeated exposure to the cold room created alterations in gene expression, we minimised this exposure by never collecting flies from a given cage more than three times over the course of the experiment. No more than two flies were collected from a cage on a given day, for three reasons. Firstly, we wanted to make sure that flies were obtained from a range of different cages in order to avoid issues of results being confounded by cage of origin (such as an infection specific to one cage of flies). We therefore never obtained more than six flies from a single cage over the course of the experiment. Second, we wanted to minimise the time that samples spent at temperatures above -20°C after death, limiting the number of samples that could be collected in a single sitting. Third, all flies were collected at the same approximate time of day (morning) to minimise gene expression variation due to circadian cycles [27], limiting the number of collections that could be performed on the same day.

RNA was extracted from individual fly heads. Single heads contain enough material for RNA sequencing and can easily be removed without the need for precise dissection, providing a quick and convenient tissue for sampling. We avoided the abdomen because of the important effect that sex and the ovarian cycle would have on gene expression in these tissues. RNA extractions were performed using PicoPure kits (Arcturus), increasing the volume of extraction buffer and alcohol to 120μl. cDNA libraries were prepared using SuperScript III Reverse Transcriptase (Invitrogen).

### 2.2. Sequencing

**Fig 1:**
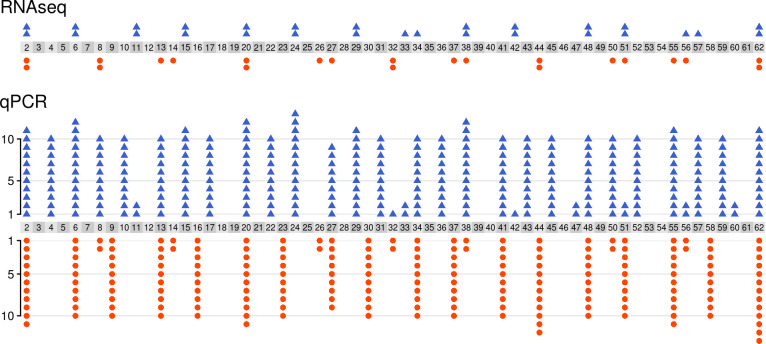
Number of samples used for RNAseq (top; total = 50) and qPCR (bottom; total = 498), split by age category (2–62 days old). Individual female and male flies shown as blue triangles and orange circles respectively.

cDNA libraries from 22 male and 28 female individual flies ranging in age from 2 to 62 days post-eclosion (Fig 1, S1 Data) were sent to the Liverpool Centre for Genomic Research (CGR) for 150bp paired-end sequencing on an Illumina HiSeq 4000 sequencer. Strand-specific library preparation was performed using NEBNext poly A selection and Ultra Directional RNA library preparation kits, producing an average of 23.8 million reads per sample. Reads were then trimmed as part of the CGR’s genomic pipeline using Cutadapt version 1.2.1 [28] with option -O 3 to remove Illumina adapter sequences, and Sickle version 1.2 (https://github.com/najoshi/sickle/releases/tag/v1.2) with a minimum window quality score of 20. Reads shorter than 20 bp after trimming were removed and subsequently unpaired reads were excluded. Data were quality checked using FastQC [29] before analysis.

### 2.3. RNAseq analysis

Trimmed reads were aligned to the GmorY1.9 genome using STAR aligner version 2.7.0 [30] using the—quantMode GeneCounts option to obtain mapping counts for each gene.

Differential expression analysis was performed using the R package *EdgeR [31]*, with library size normalisation performed using Trimmed Mean of M-values [32] and dispersion calculated with trended and tag-wise estimates. Genes with fewer than 10 reads across all 50 samples were excluded from the analysis. All plotting figures show expression measured as reads per million reads (RPM) from normalised library sizes. Association of gene expression with age and sex was tested using generalised linear modelling (glm) implemented in *edgeR*, with age coded as a continuous variable and sex as a categorical variable. Preliminary analysis found little evidence of an important effect of the number of times a colony was exposed to the cold room on gene expression, but there was a significant effect of the number of days since flies had received a blood meal (S1 Text). We therefore controlled for days since receiving a blood meal by including it as a fixed continuous factor in the glm. False discovery rate control was set at 1% using the R package *fdrtool* [33].

Gene clustering analysis was performed with the *WGCNA* package in R [34], using the normalised read counts generated by e*dgeR* and keeping only the 5000 genes with the highest variance in expression. We used the hybrid module merging algorithm with a deep split value of 4, a minimum cluster size of 30 and a power parameter of 8, followed by module merging using the absolute value of the correlation coefficient between eigengenes as a distance matrix and a merging threshold of 0.2.

Prediction of age based on normalised read counts from the RNAseq data was performed using lasso regression implemented with the *glmnet* package in R [35]. As the aim was to find genes with consistently high predictive value for age, we explored a range of lasso parameters. This exploratory procedure is recorded in detail in the R script “02_lasso.r” provided on GitHub (https://github.com/EricRLucas/TsetseAgeMarkers).

### 2.4. Primer design and qPCR

Based on the results of the RNAseq analysis, 16 genes were short-listed to be tested as qPCR markers of age in *G*. *morsitans*, with two further genes being identified as suitable housekeeping genes for our purposes (i.e.: showed minimal variation in expression in the conditions included in our study and no evidence of association with age). Primers were designed for these genes based on the GmorY1.9 genome using NCBI Primer blast [36]. Where possible, amplicons were designed to span exon junctions. Based on testing amplification efficiency using 1:3 serial dilutions, the 10 best primer pairs for age-predictive genes, and the two primer pairs for housekeeping genes, were kept for use in the study and applied to 499 samples (298 females and 201 males), including 44 of the samples used for RNAseq (the remaining 6 samples had too little cDNA left to be included in the qPCR study). One of the samples failed to produce a Ct value for several genes and was therefore excluded from subsequent analysis, leaving 498 samples (Fig 1). All primers used in this study are listed in S2 Data.

qPCR was run on a AriaMX RealTime PCR instrument in a total volume of 20 μl, containing 10 μl of SYBR 2x MM, 1.2 μl of forward primer (5μM), 1.2 μl of reverse primer (5μM), 6.6 μl of nuclease-free water and 1 μl of genomic DNA. Reaction conditions: one cycle of 95°C (3 minutes), 40 cycles of 95°C (10 seconds) and 60°C (10 seconds), one cycle of 95°C (1 minute), 55°C (30 seconds) and 95°C (30 seconds, 5 seconds soak time).

Missing raw Ct values for age-predictive genes (where the signal never reached the threshold even after 40 cycles) were replaced with the maximum value of 40. ΔCt values were calculated using the mean Ct of the two housekeeping genes. Where Ct values were missing for either housekeeping gene, normalisation was impossible and the normalised aging gene value was recorded as missing (NA). All samples were run in two technical replicates and the final ΔCt was taken as the mean of the two replicates. Gene GMOY005321 consistently showed variable ΔCt values between technical replicates, possibly due to low expression of this gene, and these values were kept unchanged. For all other genes, any gene-sample combinations whose ΔCt differed by more than 1 between technical replicates were rerun for a third technical replicate, along with both housekeeping genes, providing a third ΔCt. In most cases, this third ΔCt was very close to one of the first two and very different from the other, indicating which of the first two technical replicates was wrong. The final ΔCt was thus taken as the mean of the third replicate and whichever of the first two replicates it was closest to.

### 2.5. Predicting tsetse age from qPCR data

Machine learning predictions of tsetse age from qPCR data were performed using the *caret* package in R (https://cran.r-project.org/package=caret). The ΔCt values for each of the 10 study genes were used as continuous predictor variables, and sex was included as a categorical predictor variable since some of the genes showed sex-dependent expression. Samples were randomly split into training set (75% of samples) and test set (25% of samples), stratified by sex and age to ensure equal representation of these two variables in the two sets. Due to rounding of sample numbers within each stratification layer, the final numbers in the train and test sets were 380 (76%) and 118 (24%) samples respectively. Model training was performed using three rounds of 10-fold cross-validation. For regression models, whose aim is to estimate age as a continuous variable, partial least squares regression (PLS), random forest and extreme gradient boosting (XGB) models were all trained on the data and their predictive accuracies compared. Categorical models were trained to categorise individuals into ≤15 and >15 days old. Simple decision tree, random forest and XGB models were compared for these categorical models.

The minimum number of expression markers (genes) required to obtain accurate predictions of age was determined by training the models with different numbers of loci. For each of the random forest and XGB models, the ten genes were ranked according to their variable importance in the full model training described above (sex was found to have a variable importance of 0 in both cases, and was therefore excluded from these models). The models were then trained with all ten genes, the top nine genes, the top eight genes, and so on. For each set of genes, 20 models were trained with a different random split of training and test sets, to account for stochastic variation in model accuracy.

All statistical analysis was conducted in R version 3.4.4 [37]. Analysis scripts are available on GitHub (https://github.com/EricRLucas/TsetseAgeMarkers).

## 3. Results

**Fig 2:**
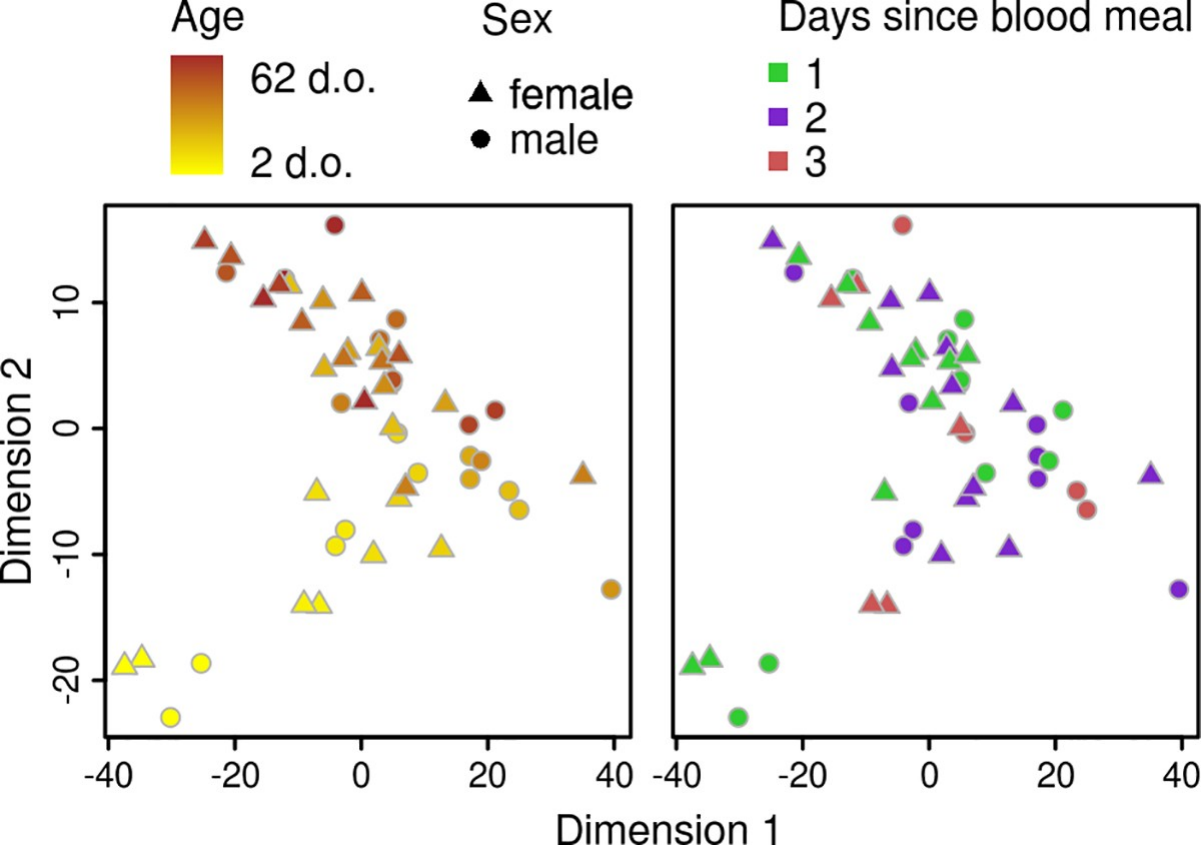
Gene expression clusters primarily by age. Principal component analysis of RNAseq data, coloured by age (left) or days since blood meal (right).

We collected 301 female and 204 male *G*. *morsitans* flies of known age from laboratory colonies, ranging in age from 2 to 62 days old. An initial RNAseq analysis of 28 female and 22 male samples showed that gene expression in these samples was primarily affected by age, rather than sex or days since last blood meal (Fig 2 and Fig A in S3 Text), although this was primarily due to the strong changes in gene expression found during the first 15 days of life, with older individuals clustering primarily by sex (Fig B in S3 text). Gene clustering analysis similarly showed that the largest cluster of correlated genes was one that changed strongly with age, particularly at young ages, with little effect of sex (S2 Text).

**Fig 3:**
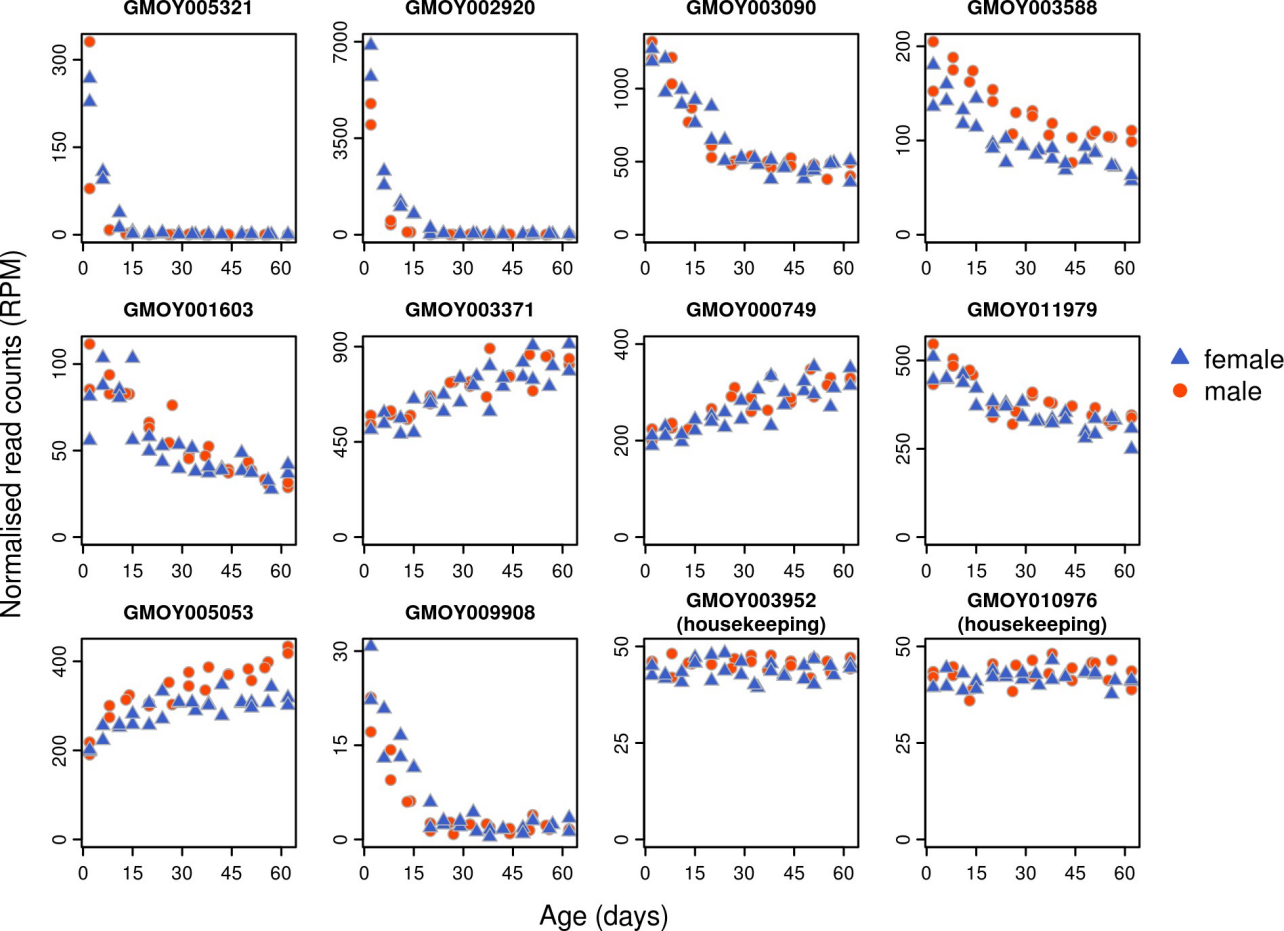
Expression of ten age-related genes and two housekeeping genes from RNAseq data, ordered according to the variable importance in the XGB model (Fig 4). Very strong early-age expression changes in some genes (eg: GMOY005321, GMOY002920) allow good discrimination among young individuals, but show little change in later life. Genes with continuous changes (eg: GMOY003371, GMOY000749) are more gradual and offer more consistent, but less powerful, discrimination at all ages.

**Fig 4:**
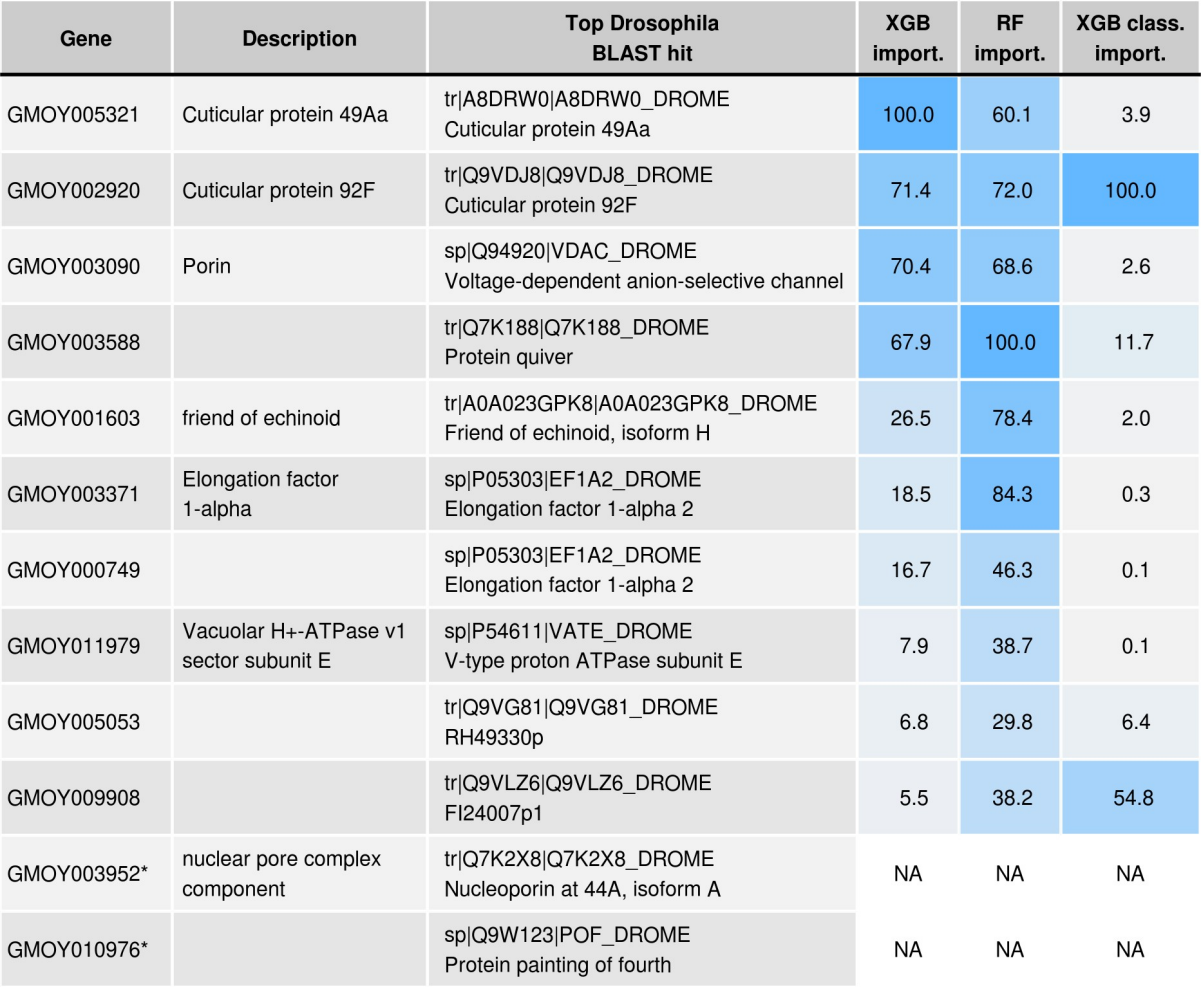
Ten age-related genes and two housekeeping genes (denoted with *) were used for qPCR analysis. Gene descriptions are taken from the Contig names in the GmorY1.9 proteome, downloaded from www.vectorbase.org/downloads on 2^nd^ of March 2019. Top *Drosophila* BLAST hits obtained by blasting the GmorY1.9 proteome against the *D. melanogaster* swissprot proteome. Variable importance of each gene shown for XGB, random forest (RF) and XGB classifier models trained with all predictor variables.

We identified a set of genes that was likely to provide strong age prediction by looking for genes that: 1. Were strongly correlated with age, or 2. consistently performed well in prediction of age using lasso regression and 3. where possible, belonged to different gene clusters as defined by weighted gene network clustering analysis. We particularly looked for genes showing strong expression changes in older individuals by identifying the genes most differentially expressed when considering only individuals older than 15 days, but even these showed relatively slight changes with age compared to some of the changes seen in the first 15 days of life (Fig 3 and Fig C in S3 Text). Using our criteria, and after testing qPCR primer efficiency, we manually picked 10 genes associated with age, and 2 genes with very little variation across samples to serve as housekeeping genes (Figs 3 and 4).

We obtained qPCR measurements of expression for these genes from 297 females and 201 males (Fig 1). As expected, expression of all 10 age-related genes was strongly correlated with age (Fig D in S3 Text) and with the RNAseq data (Fig E in S3 Text). Principal component analysis of these age-related genes showed that age dominated the first principal component of the data. In particular, samples clustered strongly into those younger and older than 15 days (Fig F in S3 Text)

**Fig 5:**
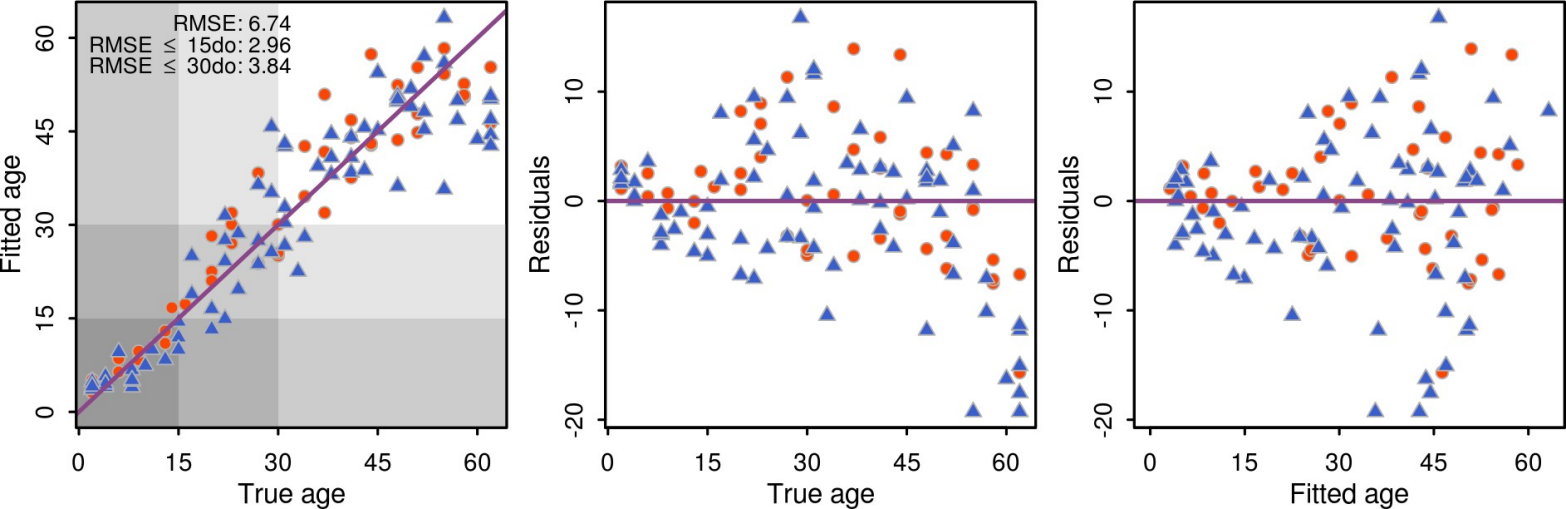
Prediction accuracy of the XGB model was highest (RMSE lowest) for individuals under 15 days old (2.96), and highest when all individuals were considered (6.74). Females are shown as blue triangles and males as orange circles. Purple line shows idealised perfect prediction.

The qPCR expression data produced strong overall predictions of age, with predictions being much more accurate in young flies (15 days or younger) compared to older flies. For regression models, PLS provided the poorest predictions of age, while random forest and XGB models performed equally well (Fig 5 and Fig G in S3 Text). Taking the XGB model as an example, the overall root mean squared error (RMSE) for the final model was 6.74 days, but was 2.96 for individuals ≤15 days old. Variable importance for each gene in the random forest and XGB models are shown in Fig 4. Training the model separately for males and females did not improve prediction accuracy (Fig H in S3 Text).

Models also performed well at classifying samples into age categories of ≤15 and >15 days old (Fig I in S3 Text). The XGB model performed best in this task, accurately classifying 117 out of 118 samples in the test set.

**Fig 6:**
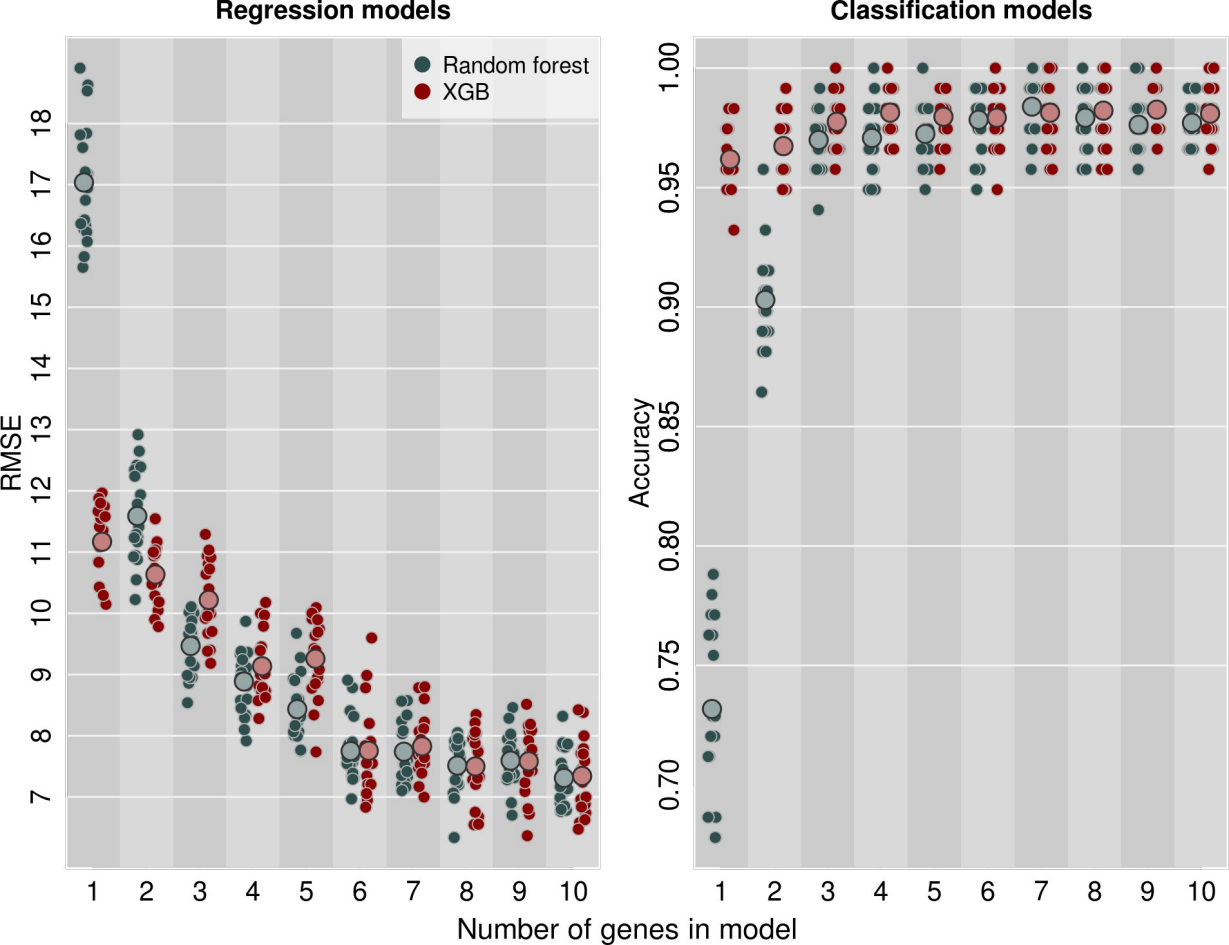
Predictive power of XGB and random forest models plateaus after the top 6 genes are included in the models (left). Accuracy of classification models plateaus after top 3 genes are included, with >95% accuracy achievable with only two genes (right). Small points show models run on independent test-train splits of the data (20 replicates per gene number); large points show the mean for each category. Points are jittered on the x axis to show overlapping data.

For both the random forest and XGB regression models, prediction accuracy showed little decrease when the variables of least importance were dropped from the models (Fig 6). In both cases, accuracy remained comparable to that with all 10 genes when only 6 genes were included, with RMSE changing from 7.3 to 7.7 (random forest) or from 7.3 to 7.8 (XGB). In contrast, when moving to 5 genes instead of 6, RMSE changed from 7.7 to 8.4 (random forest) or from 7.8 to 9.3 (XGB). Interestingly, the same 6 genes proved to be sufficient for both model types (GMOY005321, GMOY002920, GMOY003090, GMOY003588, GMOY001603, GMOY003371). For the classification models, even fewer genes were needed (Fig 6), with just two genes being sufficient for XGB classification accuracy consistently better than 95% (GMOY002920, GMOY009908).

## 4. Discussion

We have identified a set of gene expression markers that can be used to predict the age of *G*. *morsitans* tsetse flies in the laboratory. Importantly, this method can be applied to both males and females, providing accurate estimates of age in male tsetse. This is particularly important since not only do both male and female tsetse flies transmit trypanosomes, but males appear to be more likely to develop transmissible infections [15,20]. Our genetic markers were also unaffected by time since an individual’s last blood meal, making them more robust for use on wild-caught individuals, where such factors cannot be controlled.

Gene expression, like nearly all age-grading methods, is a measure of progression along some physiological trajectory. Thus, the predicted value is physiological age rather than chronological age, the former being dependent on the developmental rate of the individual while the latter is a strict measure of time elapsed since some defined notion of birth [38]. The correspondence between physiological and chronological age will thus depend on any factor that affects the developmental rate of the organism. In order to best identify markers that changed with age, we minimised the influence of such factors in our experiment by endeavouring to keep them fixed. For example, temperature and humidity were constant in our rearing conditions, and all samples were collected around the same time of day, leaving the possibility that these factors may yet influence the expression of our markers. Of course, in field conditions, these conditions will fluctuate, as will other potentially influential variables such as trypanosome infection, resource availability, circadian rhythms, stress and, in particular, seasonal and climatic fluctuations, which could affect expression directly or through their influence on developmental rate. Further work is required to test the applicability of the markers described here in field conditions. Before application as a monitoring tool, it will also be necessary to calibrate the model on a field population, and an important additional question to answer will be how valid that calibration is between field sites. Testing the accuracy of training the predictive model in one field site and applying it to other sites will indicate whether these markers can be used widely without re-calibration. If so, this will greatly improve the ease with which this tool can be applied.

The parameter perhaps most likely to affect the rate of development is environmental temperature. Mean daily temperatures fluctuate seasonally in the natural range of Glossina [21], and these fluctuations are known to affect developmental rate, for example decreasing the inter-larval period by around 0.35 days per degree [39]. If temperature or other environmental factors do affect the age-related change in expression, our markers will still be effective if the relationship between these factors and expression is determined, and the factors themselves are recorded over the study period. This would allow chronological age to be recovered from the expression-based estimates of physiological age. We also note that it is not yet clear whether chronological or physiological age is the most important parameter to estimate. For example, if increased developmental time leads to more frequent blood-feeding and / or shorter parasite maturation time, then flies will be infective at younger chronological age. Physiological age may then be the more important parameter.

Like other methods for estimating the age of vectors, prediction accuracy decreases at older ages [8,9,25,40–43]. In our data, this was because the change in expression with age was much greater in younger compared to older individuals, suggesting that the overall physiology of tsetse changes slowly after a certain life stage, and that there is thus little to detect that can be used for age grading. Although we found genes that continued to change in older ages, the rate of change relative to the variance within age groups was not sufficient to achieve the same prediction accuracies as found in younger individuals. While it is likely that more accurate old-age predictions would be achievable using whole-transcriptome methods such as RNAseq, this is too costly to be applied at the scales required for training predictive models. In mosquitoes, spectroscopy-based methods used to estimate age initially suffered from a similar loss of precision at older ages [9,43–45], but recent studies using machine learning prediction methods have improved prediction accuracies [46,47]. Whether similar performance can be achieved with tsetse should be explored.

The best estimates of maximum lifespan for *G*. *m*. *morsitans* in the field come from a mark-release-recapture study in which hundreds of newly-emerged tsetse were released and recapture attempts made over the course of six months [21,48]. Results indicated that around 30% of females survived for 60 days, and 10% survived for 110 days. In contrast, 10% of males survived to 30 days and 2% survived to 40 days. Thus, particularly for females, it would be advantageous to develop or improve age-grading tools that are accurate beyond the ages that have successfully been achieved so far.

While we used ten genes in our study, we found that using only the six genes most predictive of age still provided high prediction accuracy, and only two genes were needed for classifying individuals into age groups of ≤15 and >15 days old. By removing four genes from the analysis, qPCR time and costs can be reduced by 1/3 (eight qPCR reactions per sample instead of twelve), while removing eight genes will reduce costs by 2/3. We thus suggest that further studies testing the applicability of these markers in the field restrict themselves to either six or two genes, depending on how precisely age needs to be estimated. Such studies are needed to determine the applicability of these markers in the field, but it would also be interesting to measure the expression of these genes in age-controlled samples of other species of tsetse to determine whether these markers have widespread applicability. Once the field applicability of these markers is confirmed, the technique can be rolled out in the context of monitoring of tsetse control campaigns by comparing the age distribution before and after interventions to confirm that a resulting shift in the population age distribution is observed. In particular, in the wake of a 100% effective campaign, no flies older than the start of the campaign should be found. The resulting data on age structure both before and after control campaigns can then also be used to inform epidemiological models of trypanosomiasis transmission.

In conclusion, our study provides a new method for estimating the age of tsetse flies which does not require specialist dissection skills and can be applied to males. Testing of the applicability of these markers in the field in now required, and the problem remains of finding methods for more accurately estimating age in older individuals. This may involve identifying senescent changes whose rate is steady and consistent enough to be generalisable to any individual in the population.

## Supporting information

S1 Data Samples table. (XLS)

S2 Data Primer information table. (XLS)

S1 Text Preliminary RNAseq analysis details. (PDF)

S2 Text Gene clustering analysis results. (PDF)

S3 Text Supplementary figures. **Fig A:** Principle component analysis (PCA) of RNAseq data. Fig B: Hierarchical clustering of samples from RNAseq data reveal that young individuals (< 15 days old) cluster together, with older individuals clustering by sex. Fig C: Expression changes with age for the 6 genes most strongly differentially expressed by age when only individuals older than 15 days were included in the model. Fig D: Correlation of qPCR measures of gene expression against age for the ten genes chosen as age markers. Fig E: Expression measured by qPCR and RNAseq were highly correlated in the samples in which both techniques were used. Fig F: PCA of samples based on qPCR measurements of expression of the 10 age-related genes. Fig G: Age prediction performance of PLS, random forest and XGB regression models. Fig H: Age prediction performance of random forest and XGB regression models trained separately on females and males. Fig I: Accuracy at classifying samples into age groups of ≤ 15 and > 15 days old was 99% for the XGB classification model, 98% for the random forest and 97% for the decision tree.
(PDF)
