# Supplementary material for: A gene expression panel for estimating age in males and females of the sleeping sickness vector Glossina morsitans
Source: PLoS Negl Trop Dis. 2021 Sep 23;15(9):e0009797. doi: 10.1371/journal.pntd.0009797 (PMC8491940; doi:10.1371/journal.pntd.0009797)
Supplement: S2 Text — (PDF) [file pntd.0009797.s004.pdf]

# A gene expression panel for estimating age in males and females of the disease vector *Glossina morsitans*

Eric R. Lucas, Alistair C. Darby, Stephen J. Torr, Martin J. Donnelly

## Electronic Supplementary Material

## S2 Text

## Results of WGCNA analysis

Thirty modules were identified by Weighted Gene Network Clustering Analysis (WGCNA), many of which showed signs of change with age (Fig. **S2.1**). The largest module in terms of number of genes (MEturquoise module, Fig. **S2.1**) displays very strong correlation with age, particularly at young ages, and little or no difference between sexes. In fact, when simply looking at the list of genes most strongly correlated with age, the large majority belong to this module. In order to avoid our panel containing too much redundant information, we consciously aimed to include genes from other modules (Fig. **S2.2**).

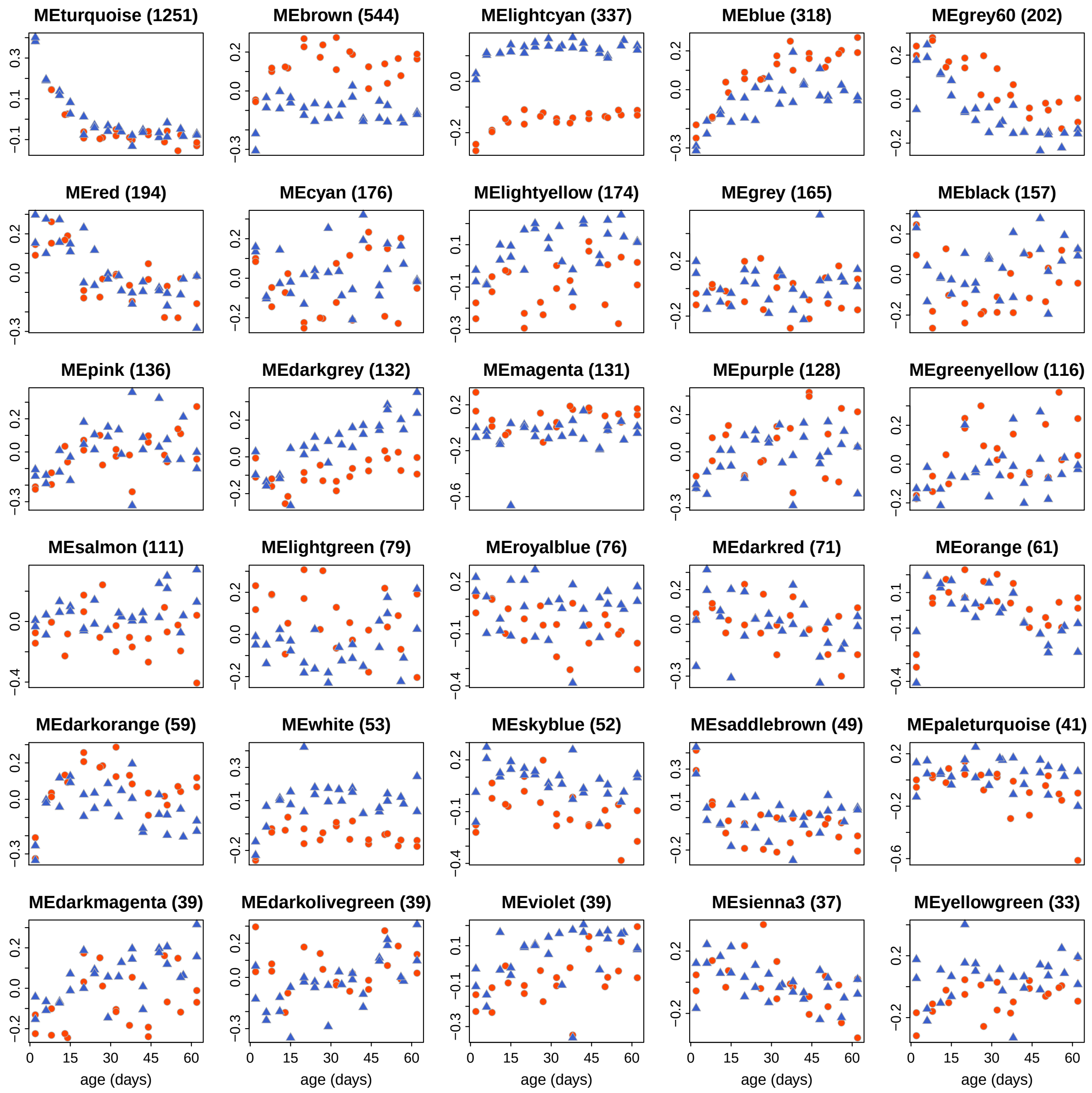


**Fig. S2.1**: Each plot shows represents one of the modules determined by WGCNA, plotting the value of that module's eigengene (combination of all genes that are members of the module) for each of the samples. Blue triangles are females; orange circles are males. The title of each plot shows the name of the module (assigned as a colour by WGCNA) followed by the number of genes in the module in brackets. Y axes are in arbitrary units of the eigengenes. Modules are sorted in order of number of genes contained within.

| GMOY000749 | GMOY001603 | GMOY002920 | GMOY003090 | GMOY003371 | GMOY003588 | GMOY005053 | GMOY005321 | GMOY009908 | GMOY011979 |
|---|---|---|---|---|---|---|---|---|---|
| MEturquoise | MEgrey60 | MEturquoise | MEred | MEturquoise | MEgrey60 | MEturquoise | MEturquoise | MEturquoise | MEgrey60 |

**Fig. S2.2**: Module assignment of the 10 genes chosen for the final qPCR panel. Names refer to modules in Fig. **S2.1**.
