# Supplementary material for: A gene expression panel for estimating age in males and females of the sleeping sickness vector Glossina morsitans
Source: PLoS Negl Trop Dis. 2021 Sep 23;15(9):e0009797. doi: 10.1371/journal.pntd.0009797 (PMC8491940; doi:10.1371/journal.pntd.0009797)
Supplement: S3 Text — Fig A: Principle component analysis (PCA) of RNAseq data. Fig B: Hierarchical clustering of samples from RNAseq data reveal that young individuals (< 15 days old) cluster together, with older individuals clustering by sex. Fig C: Expression changes with age for the 6 genes most strongly differentially expressed by age when only individuals older than 15 days were included in the model. Fig D: Correlation of qPCR measures of gene expression against age for the ten genes chosen as age markers. Fig E: Expression measured by qPCR and RNAseq were highly correlated in the samples in which both techniques were used. Fig F: PCA of samples based on qPCR measurements of expression of the 10 age-related genes. Fig G: Age prediction performance of PLS, random forest and XGB regression models. Fig H: Age prediction performance of random forest and XGB regression models trained separately on females and males. Fig I: Accuracy at classifying samples into age groups of ≤ 15 and > 15 days old was 99% for the XGB classification model, 98% for the random forest and 97% for the decision tree. (PDF) [file pntd.0009797.s005.pdf]

A gene expression panel for estimating age in males and females of the disease vector *Glossina morsitans*

Eric R. Lucas, Alistair C. Darby, Stephen J. Torr, Martin J. Donnelly

Electronic Supplementary Material
S3 Text (Supplementary figures)

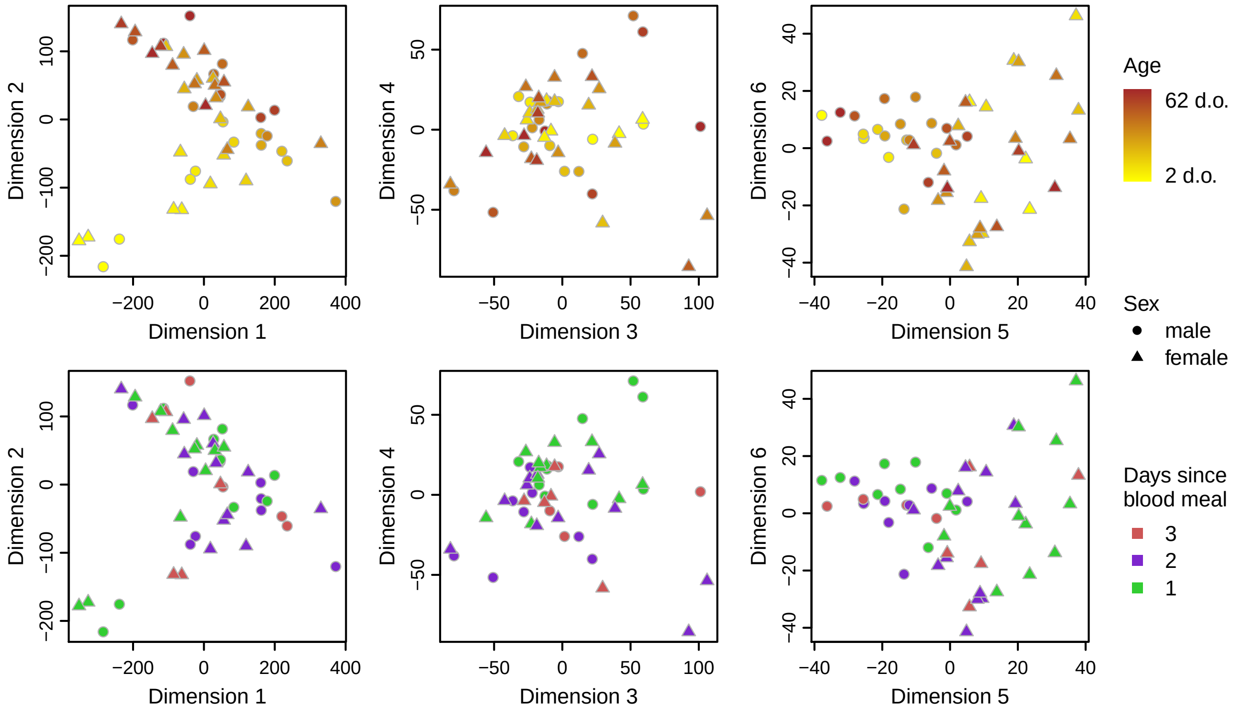

Fig. A: Principle component analysis (PCA) of RNAseq data, coloured by age (top) or days since blood meal (bottom).

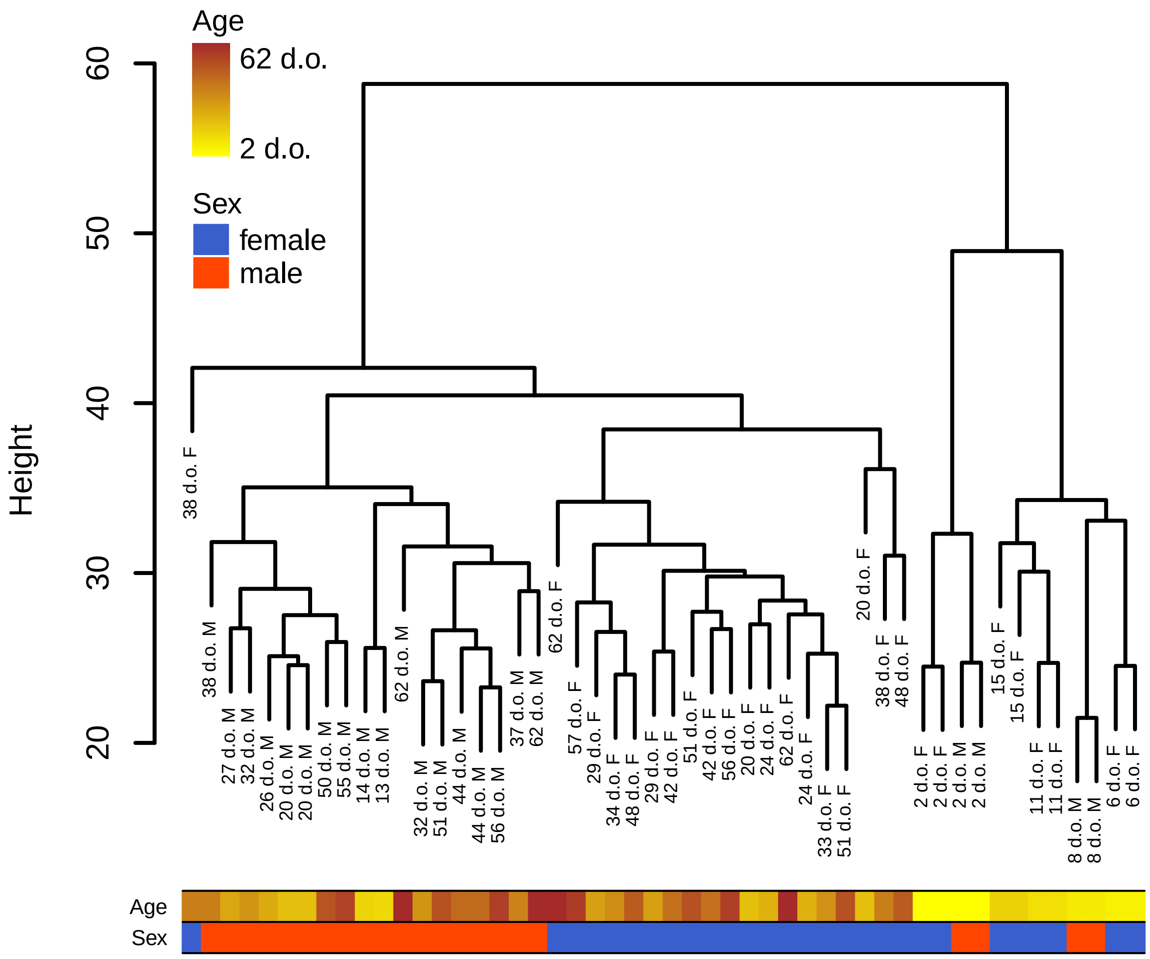

Fig. B: Hierarchical clustering of samples from RNAseq data reveal that young individuals (<15 days old) cluster together, with older individuals clustering by sex.

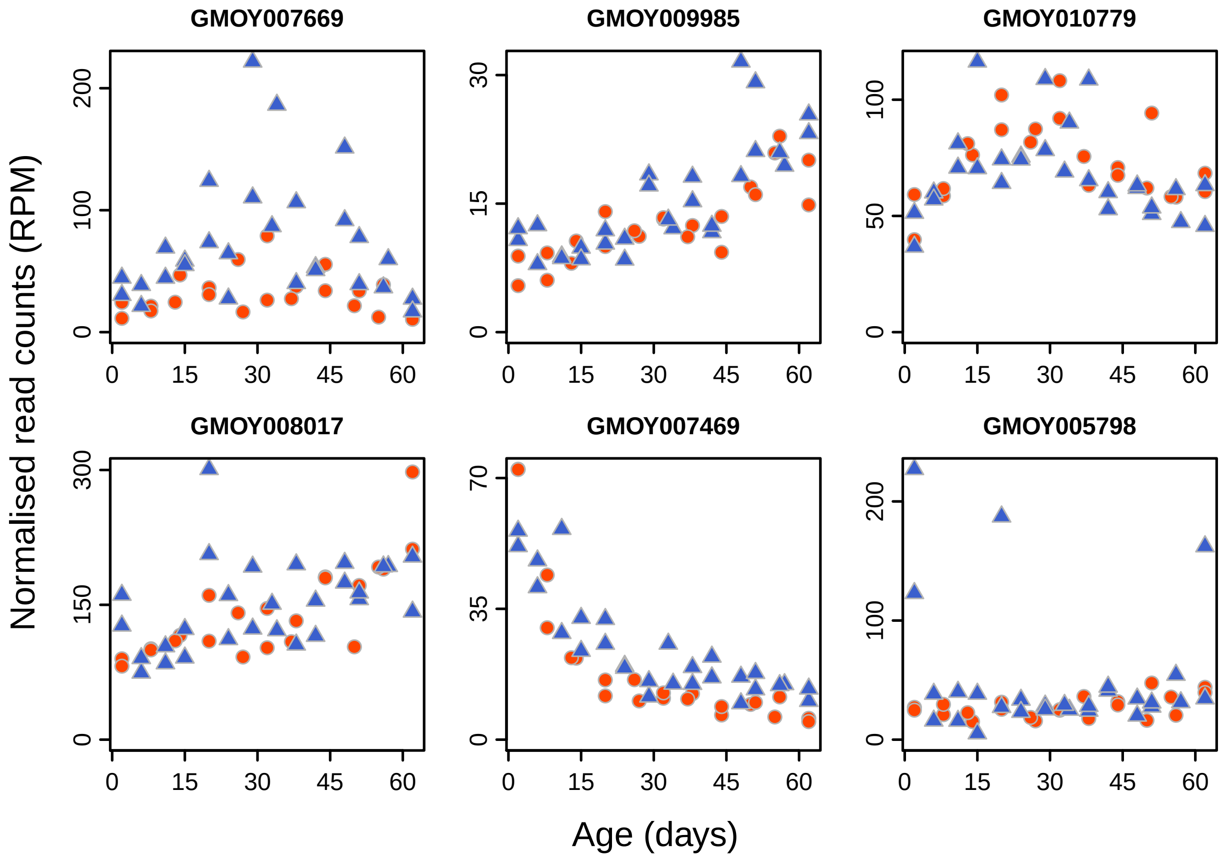

Fig. C: Expression changes with age for the 6 genes most strongly differentially expressed by age when only individuals older than 15 days were included in the model. Females shown as blue triangles; males shown as orange circles.

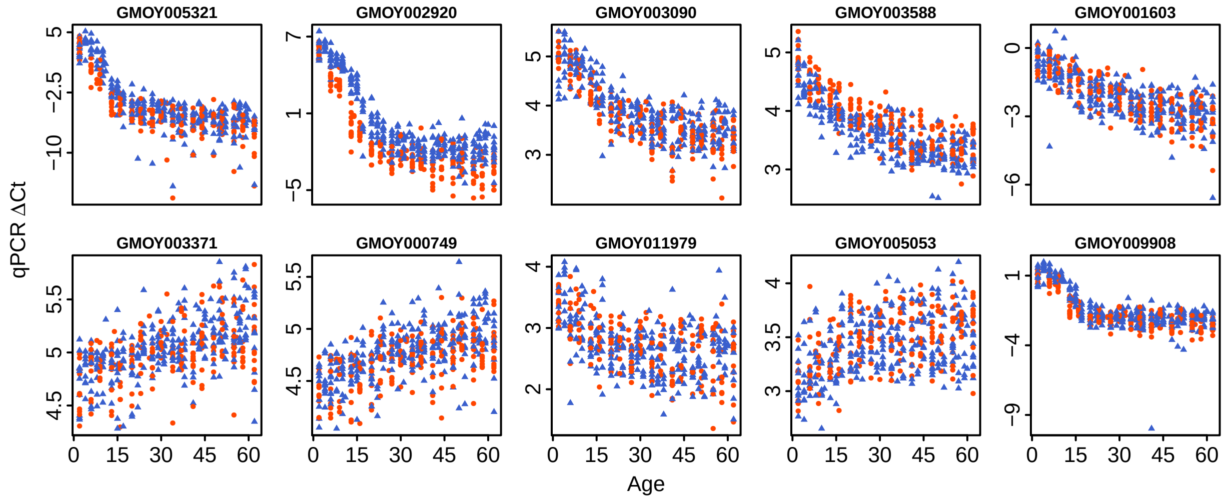

Fig. D: Correlation of qPCR measures of gene expression against age for the ten genes chosen as age markers. Females shown as blue triangles; males shown as orange circles.

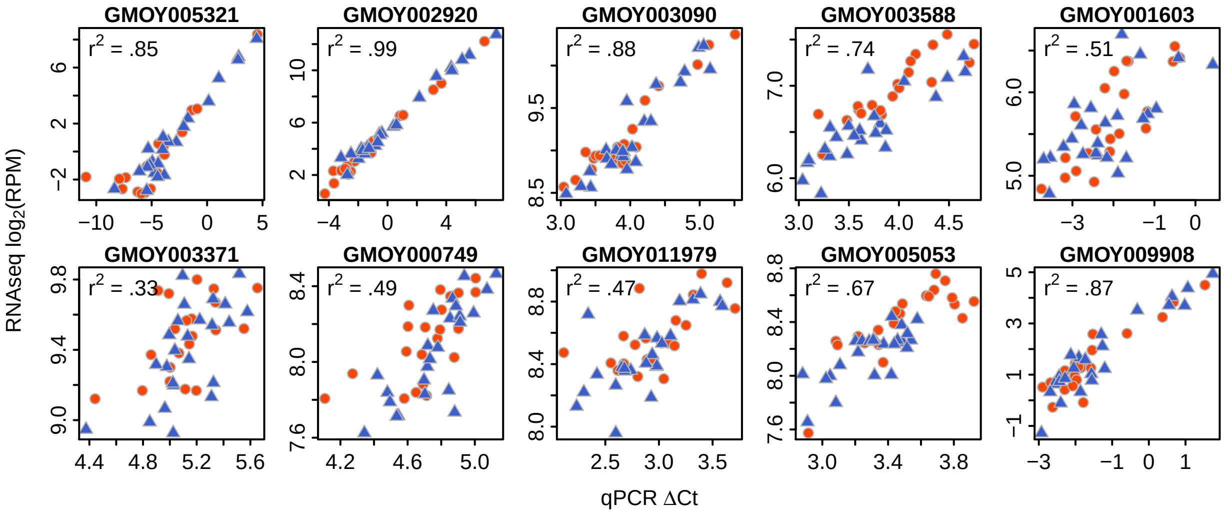

Fig. E: Expression measured by qPCR and RNAseq were highly correlated in the samples in which both techniques were used. Expression by RNAseq is here measured as $\log_2(\text{RPM} + 0.1)$. RPM = reads per million. 0.1 added to avoid taking logs of 0. Females shown as blue triangles; males shown as orange circles.

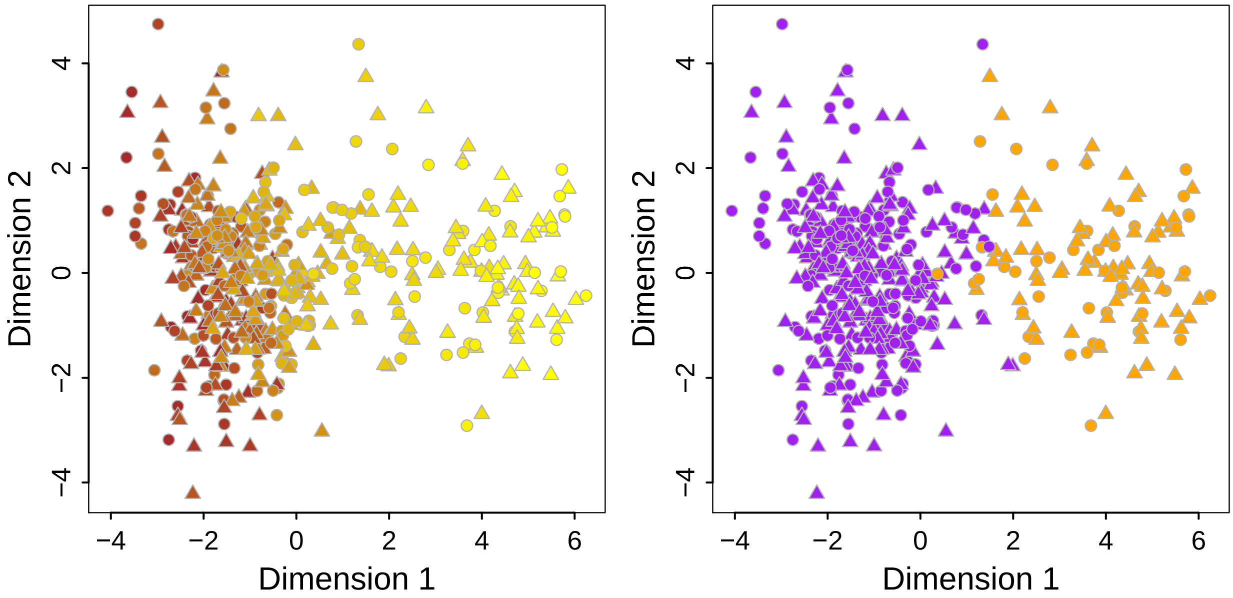

Fig. F: PCA of samples based on qPCR measurements of expression of the 10 age-related genes. Left: colour-coded continuously from 2 days old(yellow) to 62 days old (brown). Right: colour-coded categorically into ≤ 15 days (orange) and > 15 days (purple). Females shown as triangles; males shown as circles.

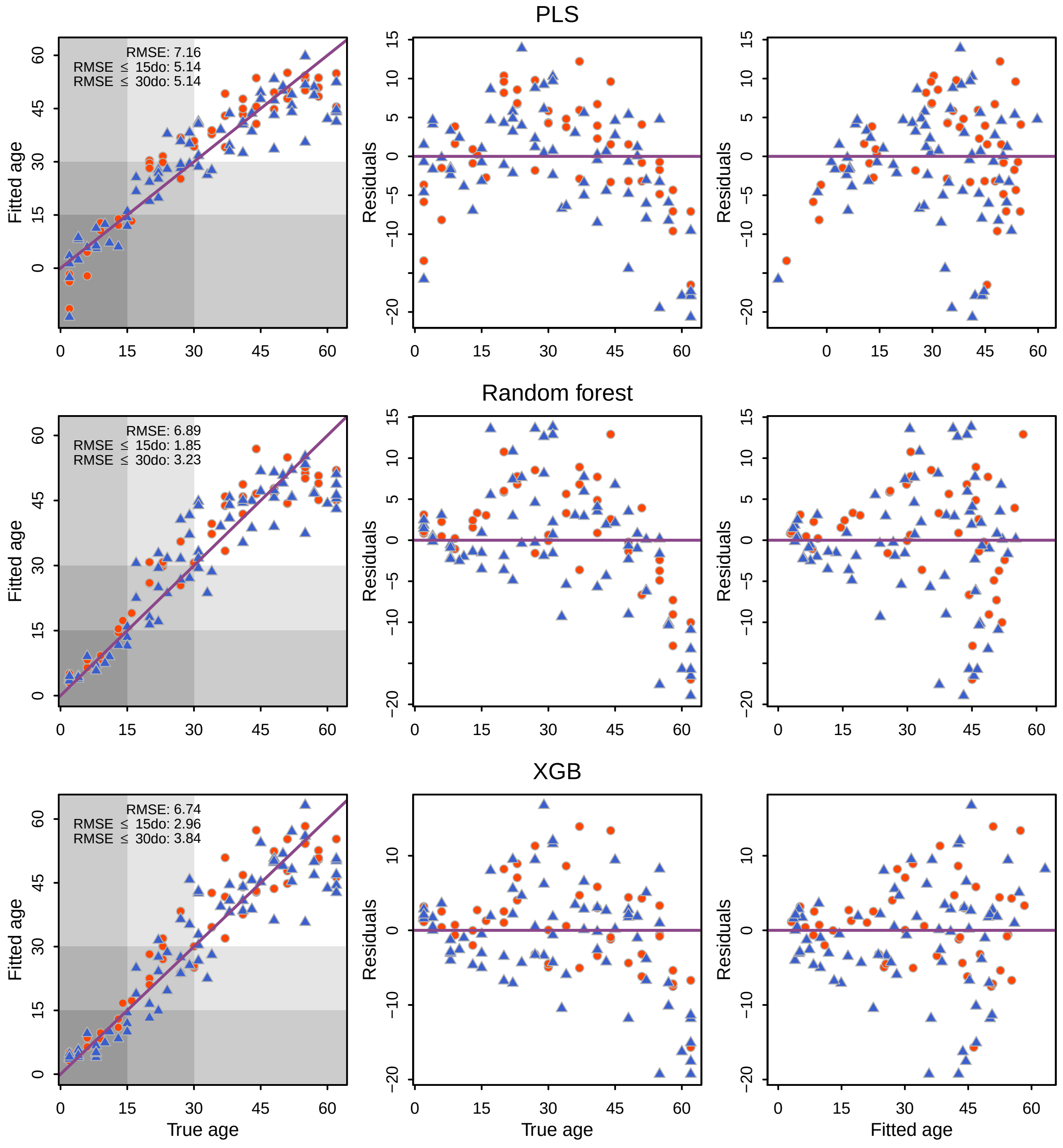

Fig. G: Age prediction performance of PLS, random forest and XGB regression models. Females shown as blue triangles; males shown as orange circles.

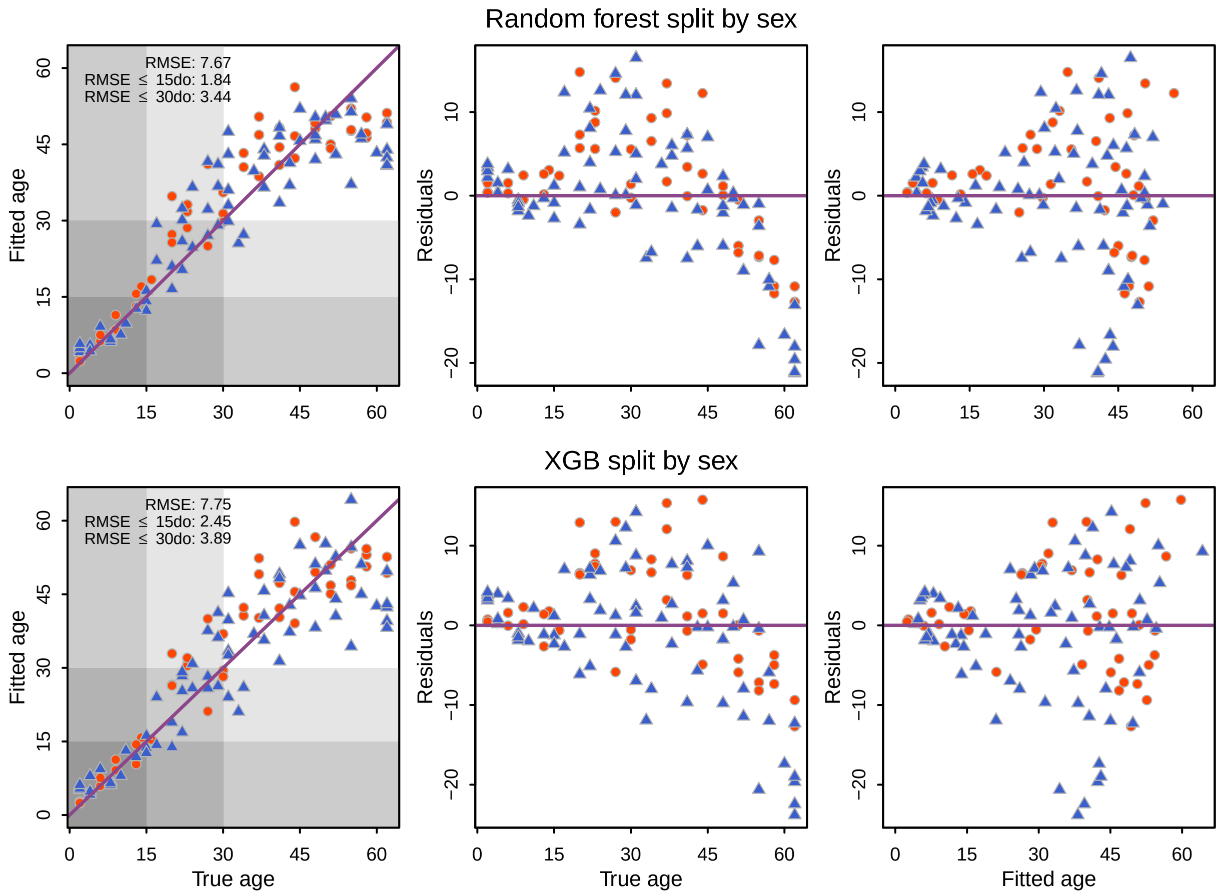

Fig. H: Age prediction performance of random forest and XGB regression models trained separately on females and males. Females shown as blue triangles; males shown as orange circles. Purple line shows idealised perfect precdiction.

		Decision tree prediction		*Random forest prediction*		*XGB prediction*	
		**Young**	**Old**	**Young**	**Old**	**Young**	**Old**
True age	**Young**	25	3	27	1	28	0
	**Old**	0	90	1	89	1	89

Fig. I: Accuracy at classifying samples into age groups of ≤ 15 and > 15 days old was 99% for the XGB classification model, 98% for the random forest and 97% for the decision tree.
